# Procalcitonin Perplexity – Prolonged Idiopathic Elevation in Pleomorphic Sarcoma: A Case Report and Review of the Literature

**DOI:** 10.7759/cureus.8215

**Published:** 2020-05-20

**Authors:** Alheli Arce Gastelum, Thomas Volberding, S. Caleb Freeman, Mary Dick, Maryam Gbadamosi-Akindele

**Affiliations:** 1 Internal Medicine, Creighton University School of Medicine, Omaha, USA; 2 Dermatology, Creighton University School of Medicine, Omaha, USA

**Keywords:** procalcitonin, sepsis, antibiotic stewardship, pleomorphic sarcoma, infectious disease, oncology, cancer, infection, antibiotic administration

## Abstract

Historically, elevations in procalcitonin (PCT) have been implicated in medullary thyroid cancer and neuroendocrine tumors. More recently, the trending of PCT has been suggested as a monitor of infection to assess the presence, clearance and eradication of infection, especially in cancer patients. Its increase serves as a marker of bacterial infections. During homeostasis it is produced by most tissues in the body at an extremely low level (<.01 ng/mL) and is often induced by bacterial endotoxins. In cancer patients additional factors influence these levels. Metastasis in particular is linked with relatively higher PCT levels.

We present a case of an afebrile patient with undifferentiated pleomorphic sarcoma who underwent 25 cycles of radiation therapy and presented one month later with elevated procalcitonin, lactic acid, and leukocytosis. All infectious work up was negative. Findings were incidental after a hospital visit for dehydration. Leukocytosis and lactic acidosis resolved after four days into the hospitalization. Procalcitonin, however, remained elevated over four months in the range of 2-5 ng/mL. The patient has no findings of metastatic disease. To our knowledge, there has never been a report in the literature describing a prolonged elevation of procalcitonin in a patient with a non-metastatic sarcoma without any signs of infection or any other underlying cause.

The elevation of PCT has been noted in patients who suffered burns, trauma, minor and major surgery, and cardiogenic shock in addition to infection. Increases have served as signs of worsening patient outcomes and elevated rate of complications. Trending PCT can help in appropriated antibiotic use as it has been shown to decrease antibiotic use by 2.4 days. PCT trends have been increasing in value making idiopathic elevations found in combination undifferentiated pleomorphic sarcoma an important addition to the literature.

## Introduction

Procalcitonin (PCT) is a 116 amino acid protein that serves as the precursor of calcitonin. In the absence of systemic inflammation procalcitonin synthesis is restricted to thyroid neuroendocrine cells and found in very low levels in the circulation (<0.1 ng/mL) [1]. However, during bacterial infection PCT synthesis is triggered by bacterial endotoxin and other inflammatory markers such as TNF alpha, IL beta, and IL 6 [2]. In comparison, viral infections are known to release IFN gamma, an inhibitor of TNF alpha thereby making PCT more specific to bacterial infections [1,2]. More recently PCT has received substantial interest as a potential marker of infection to assess the presence, clearance, and eradication of infection; predict mortality; and guide antibiotic management [2-5].

Although PCT can be specific to bacterial infection, there are various other etiologies for elevated PCT levels. Elevations in PCT have been implicated in medullary thyroid cancer and neuroendocrine tumors as well as in burns, malignancy and renal dysfunction [2,5]. As in the case of this patient, who was erroneously diagnosed with sepsis multiple times, an elevated procalcitonin can be a confounding diagnostic factor.

We present a case of an afebrile patient with undifferentiated pleomorphic sarcoma who curiously presented with elevated procalcitonin, lactic acid and leukocytosis who had a negative infectious workup. To our knowledge, there has never been a report in the literature describing a prolonged elevation of procalcitonin in a patient with a non-metastatic sarcoma without any signs of infection.

## Case presentation

NT is an 86-year-old man with a past medical history of high-grade pleomorphic sarcoma of the left axilla and back for which he finished therapy with radiation of 5000 cGy in 25 fractions, bioprosthetic aortic valve replacement, type 2 diabetes mellitus, and hyperlipidemia. He has had multiple recent hospitalizations for febrile episodes of unclear etiology and weakness during which he was found to have leukocytosis with elevated procalcitonin and lactic acid levels. Previous hospitalizations found no source of infection. In this presentation, the patient endorsed increasing generalized weakness for the past seven days and development of an unsteady gait. He was initially brought to the emergency department by his son who witnessed the patient take a staggering fall into a doorway that caused a skin tear on his forearm and an abrasion on his scalp. Patient's review of system was only positive for decreased oral intake and lack of appetite. In the emergency department, he was once again found to have leukocytosis of 14.2 k/ul, with an elevated procalcitonin of 5.1 ng/mL and lactate of 2.5 mmol/L.

On physical exam, the patient's vital signs were within normal ranges for his age. The patient was alert, oriented and cooperative. Cardiac exam revealed a normal rate and rhythm free from murmurs, rubs, or gallops. Lungs were clear to auscultation bilaterally. Abdomen was nontender and nondistended. Exam did show a firm, fixed, and non-fluctuant 10-cm mass in the left axilla and upper back. The patient also had a 2-cm abrasion on the left forehead and a skin tear on the left forearm.

**Figure 1:**
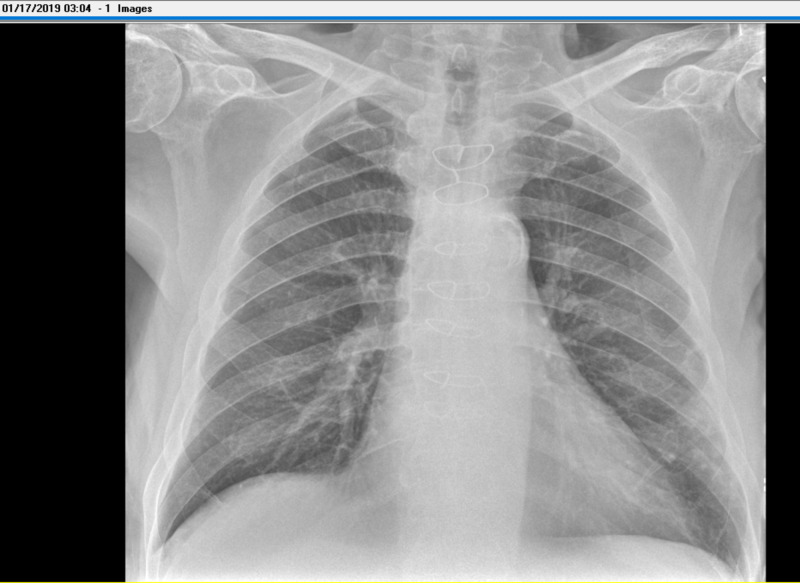
Chest X-ray Chest X-ray without acute cardiopulmonary abnormalities.

**Figure 2:**
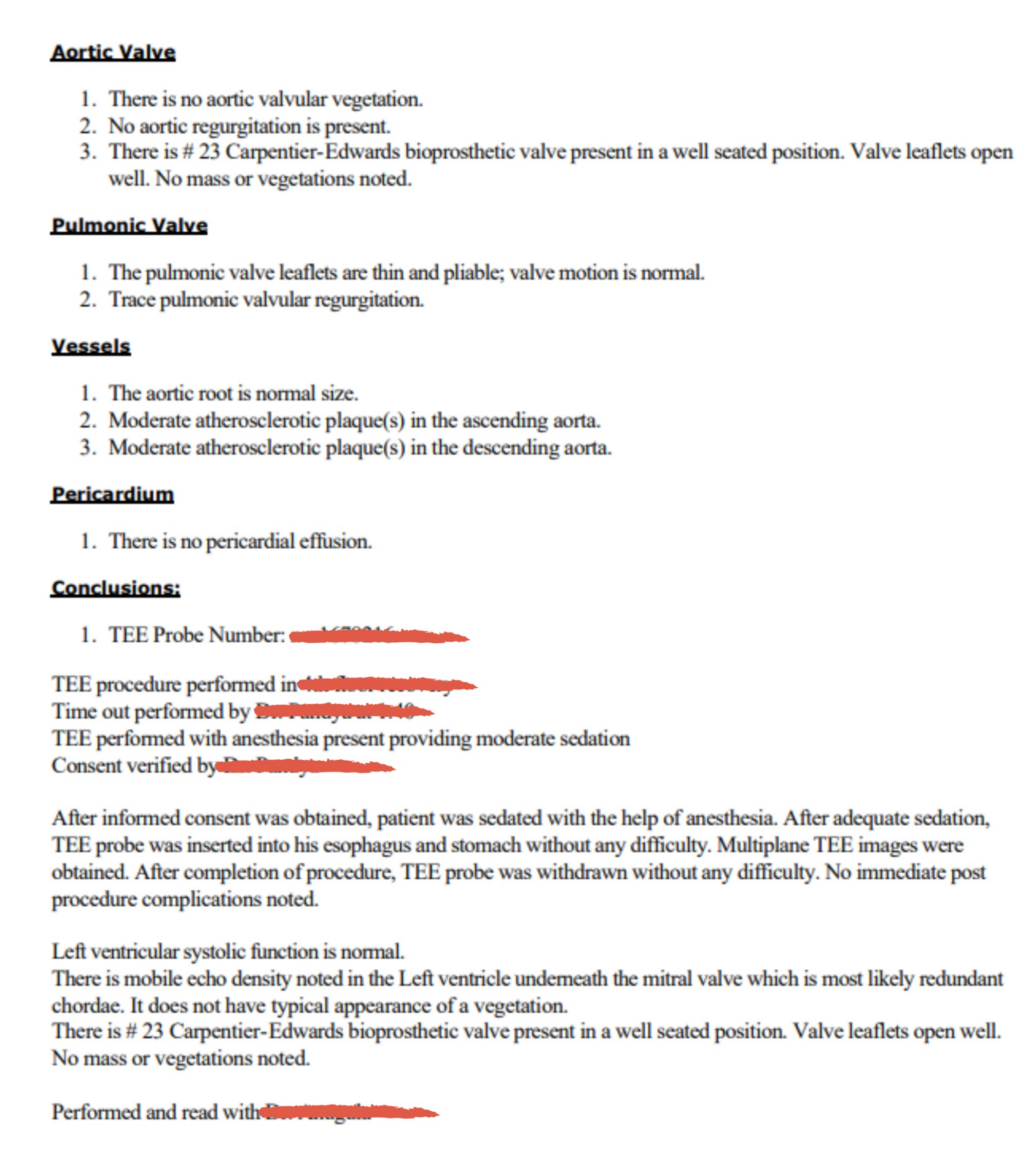
Transesophageal echocardiogram (TEE) report Transthoracic echocardiogram report with absence of vegetations.

**Figure 3:**
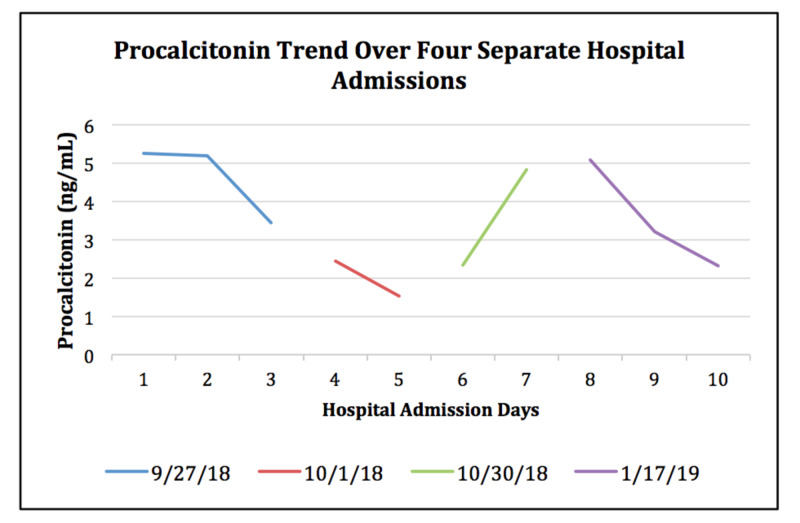
Procalcitonin trended over four separate hospital admissions from September 2018 to January 2019. Procalcitonin remained above normal reference ranges despite repetitive negative infectious workups.

Lab results on admission were notable for continued leukocytosis, hyponatremia with a sodium of 135 mEq/L, hemoglobin of 10.7 gm/dl, and mean corpuscular volume (MCV) of 81.1 fL. Platelets were at 486,000. Iron and ferritin levels taken during the prior hospitalization showed a low serum iron at 20 ug/dL with an elevated ferritin at 681 ng/mL, findings consistent with anemia of chronic disease. Chest X-ray showed no focal consolidations or effusions (Figure 1). Urine was clear and yellow. Urinalysis was free of protein, bacteria, leukocyte esterase, and nitrites. Echocardiogram taken one month prior this admission had shown a mobile echo density in the left ventricle underneath the mitral valve which was most likely redundant chordae (Figure 2). Previous hospitalizations also showed normal urinalysis and chest X-ray. Over the next several hospital days, leukocytosis and elevated lactate resolved spontaneously with intravenous fluid resuscitation and a one-day course of vancomycin and piperacillin-tazobactam. Blood cultures were repeated twice and remained negative during this admission. Procalcitonin returned to patient’s baseline, but never returned to a value within reference ranges (<0.5 ng/ml) (Figure 3). This is also consistent with previous hospitalizations, where the patient was admitted, requiring infectious workup and one to two days of antibiotics that were discontinued after failing to identify a source of infection. Infectious disease was consulted during this admission and a month prior; after evaluating all potential sources of infection, it was determined that the etiology of the patient's elevated procalcitonin was most likely secondary to malignancy.

## Discussion

The role of PCT as a biomarker of bacterial infection is well known and has been used to help guide diagnosis uncertainty in bloodstream infections. Procalcitonin is more specific for bacterial infections than other inflammatory markers, such as white blood cell count, erythrocyte sedimentation rate, and C-reactive protein [2]. In a study done by Maisel et al., the use of PCT with a cutoff point of 0.25 ng/mL was able to reduce pneumonia diagnosis uncertainty by 82% in the emergency department. In comparison, a PCT cut off point <0.29 ng/mL was able to exclude bacterial coinfection in influenza patients [3]. Furthermore, PCT can even help distinguish gram negative bacteria (GNB) vs. gram positive bacteria (GPB) [4]. In a study sampling of 1,949 patients with suspected bloodstream infections, GNB had significantly higher levels of PCT (13.8 ng/mL) vs. GPB (2.1 ng/mL) vs. fungal (0.5 ng/mL) (P < .0001) [5].

Clinical use and antibiotic stewardship

Several studies have implemented a PCT trending protocol as a guide for clinical management and aid in successful antibiotic stewardship both in and out of the ICU. In a study by Schuetz et al., PCT levels increase within 4 hours of infection and peaks between 8-24 hours, with highest levels indicating bacteremia and correlating with infection severity [6]. Compared to blood cultures which can take 24-48 hours for results, the use of PCT with a cutoff of 0.5 ng/mL can help diagnose bacterial infection quicker and allow more immediate antibiotic initiation. Early onset of antibiotic therapy is correlated with better outcomes.

Additionally, as inflammation resolves, PCT levels decline 50% every 1-1.5 days making PCT a useful tool for monitoring infection control [7]. In the ProRATA study, procalcitonin levels were checked daily, and clinicians were advised to stop antibiotics when levels were ≤0.5 ng/mL or if the level decreased by ≥80 percent from peak. By trending PCT levels, antibiotic duration can be decreased by 2.43 days, reducing chances for antibiotic resistance and unwanted side effects [6]. When bacteria are exposed to prolonged antibiotics, resistance can occur by genetic alterations in bacterial genome. Furthermore, prolonged or unnecessary use of antibiotics can lead to Clostridium difficile diarrheal infections, dermatologic rashes, electrolyte abnormalities, and cardiac arrhythmias.

Prognosis

In contrast, a non-declining PCT level despite antimicrobial therapy can indicate worsening prognosis. An increase in PCT levels from hospital admission to Day 3 was observed with statistically higher frequency in community acquired pneumonia patients with 30-day mortality [8]. Furthermore, a higher baseline PCT on admission or even a slower rate in decline of PCT has been correlated with worse clinical outcomes. Regardless of the underlying cause of infection, the TRIAGE trial done in 2017 showed that higher levels of PCT were found to be a statistically significant independent predictor of 30-day mortality, ICU admission and hospital readmission. In addition, the MOSES study helped show the importance of the rate of PCT decline in severely septic patients emphasizing that when PCT levels did not decrease by more than 80% from baseline to day 4, the 28-day mortality doubled. Therefore, PCT trending can help identify early non-responders to antibiotics and allow physicians to seek other causes of inflammation and sepsis.

Limitations

Despite the overwhelming studies advocating for the usefulness of PCT in bacteremic and septic patients, PCT as an infectious biomarker has its limitations. Procalcitonin has a sensitivity of 75% and a specificity of 79% with respect to bacteremia and sepsis and a negative predictive value of 98%. The caveat, however, is a very low positive predictive value of 17% [9]. As in the case of this patient, who was erroneously diagnosed with sepsis multiple times, an elevated procalcitonin can be a confounding diagnostic factor. In cancer patients, additional factors can influence PCT levels. Metastasis, in particular, is linked with relatively higher PCT levels. Therefore, it becomes difficult to distinguish a fever related to tumor progression from a fever caused by bacterial infection [10, 11].

PCT and cancer

PCT use in cancer is challenging due to the multiple scenarios that can lead to its elevation. PCT elevation of >0.25 ng/mL is usually seen in medullary thyroid cancer (MTC) and lung cancers with neuroendocrine components whereas an elevation of <0.25 ng/mL is seen in sarcomas, lymphomas, pancreatic cancer and renal cell disease. PCT is a useful biomarker for the diagnosis and follow-up of patients with MTC, especially when used in conjunction with calcitonin (CT); unfortunately, there is not enough data to suggest a particular threshold. Therefore, CT should continue to be the primary biomarker in MTC. The addition of PCT may be beneficial in some patient groups and may be considered an adjunct to CT in the management of patients with MTC [8].

In cases of lung cancers with neuroendocrine components, small cell lung cancers had significantly elevated PCT levels compared to pulmonary adenocarcinomas: 0.33 ng/mL versus 0.07 ng/mL. However, the diagnostic value of serum PCT levels for diagnosing carcinoma with a neuroendocrine component remains low (sensitivity 63.8%; specificity 71.9%). Another novel use of PCT in cancer patients appears to be in metastatic cancer patients, specifically to the liver. Interestingly, regardless of the primary neoplasm, PCT levels were significantly higher in patients with liver metastases versus without: 0.37 ng/mL versus 0.09 ng/mL [10].

## Conclusions

Overall, PCT elevations can be explained by multiple sources and the diagnosis of cancer does not exclude its use. This case exemplifies the importance of keeping a broad differential when utilizing PCT during the workup. Although a plethora of data advocates the usefulness of trending PCT in septic and bacteremic patients, it is important to keep infection-independent conditions in the differential. Ultimately, the use of procalcitonin algorithms should never override clinical judgment. In most trials, algorithms were often overruled by clinical judgment, underscoring the fact that the assay should be used as an adjunct to clinical judgment and not a replacement.
